# Oral mucosal lesions in pediatric patients

**DOI:** 10.3205/dgkh000536

**Published:** 2025-03-06

**Authors:** Vasudevi Ramiah, Devaki Manivannan, Karthik Shunmugavelu, Barun Kumar, Evangeline Cynthia Dhinakaran

**Affiliations:** 1 Department of Dental Surgery, Government Medical College and Hospital, Krishnagiri, India; 2 Department of Dentistry/Oral and Maxillofacial Pathology PSP Medical College Hospital and Research Institute Tambaram Kanchipuram, India; 3 Oral and Maxillofacial Surgery, Dental College and Hospital, Navi, Mumbai, India; 4 Department of Pathology, Sree Balaji Medical College and Hospital, Chrompet, Chennai, Tamilnadu, India

**Keywords:** oral mucosal lesions, traumatic ulceration, pediatric, India

## Abstract

Dental caries and periodontal diseases are the most common oral disorders, followed by other oral lesions, e.g., of the mucous membranes.

This study aimed to identify the pattern and expression of oral mucosal lesions in a pediatric population. 100 patients between 3 and 16 years of age from the department of pediatrics in a multispeciality hospital were examined. The majority of them had oral lesions (68%), with traumatic ulceration being the most common.

## Abstract (de)

Die Beurteilung der Mundgesundheit umfasst eine sorgfältige Untersuchung von Hart- und Weichgewebe. Karies und Parodontalerkrankungen die häufigsten oralen Erkrankungen sind, gefolgt von anderen oralen Läsionen.

Ziel der Studie war es, das Muster und die Ausprägung von Mundschleimhautläsionen in der pädiatrischen Bevölkerung zu identifizieren. Untersucht wurden 100 Patienten im Alter von 3 bis 16 Jahren aus der Abteilung für Pädiatrie in einem Krankenhaus mit mehreren Fachrichtungen. Die Mehrheit von ihnen hatte orale Läsionen (68%), wobei traumatische Ulzerationen am häufigsten waren.

## Introduction

Oral and dental health problems not only comprise dental caries and periodontal diseases [1], but also oral mucosal lesions (OML), which present as alterations in the soft tissue of the oral cavity. The recognition of these lesions involves thorough anamnestics and intraoral examination [2]. Oral examinations reveal variations in etiopathogenesis, clinical features, and diagnostic and prognostic characteristics [3]; furthermore, lesions may vary significantly between different geographic locations [4]. Oral mucosal lesions can be benign or potentially malignant, requiring no treatment to extensive invasive treatment [5]. To properly assess soft tissues in pediatric patients requires that the examiner possess knowledge of the normal size, shape, color, and texture of the structures concerned [3]. The presentation of lesions in oral mucosa in children can differ from adults in respect to color, size, etiology, clinical characteristics, prognosis, and treatment protocol Thus, it is important to assess the prevalence of the oral lesions in pediatric populations for appropriate management. Because literature on oral lesions among the pediatric population is scarce, the main objective of this study was to help fill the knowledge gap and identify the pattern and expression of oral mucosal lesions in a pediatric population.

## Materials and methods

The study sample included 100 patients 3–16 years old from the Department of Pediatrics in a multispeciality hospital. A thorough anamnesis was done. Demographics, chief complaint, history of presenting illness, and extra- and intra-oral examinations were performed. Intraoral examination involved assessment of hard and soft tissues as well as radiographic examination. The lesions were recorded in a structured format involving site, size, shape, color, consistency and extension. The study was done over a period of one year. The inclusion criteria were outpatients and inpatients between 3 and16 years of age. The exclusion criteria were patients who were not cooperative and patients who were unable to participate due to systemic illness. The results were collected and analyzed with chi-squared test and ANOVA using SPSS version 21.

## Results

### Demographics

The gender distribution was 52% male and 48% female. When the relationship between age and BMI were assessed, the majority of participants (54%) were normal, 26% were underweight, 11% were overweight, and 9% of the children were obese. Most of the children examined had a good OHI score. 

### Prevalence of lesions

The majority of the lesions (44%) were present in participants between 3–4 years of age (p=0.001) and the fewest were observed in the age group of 15–16 years of age. OML were present on 68% of the population. Male participants exhibited a higher number of oral mucosal lesions. 

The most common lesions observed were traumatic ulceration, followed by dentoalveolar abscess, gingivitis, and geographic tongue, accounting for 99% of the lesions, while the remaining lesions – white lesion, ankyloglossia, eruption cyst and aphthous ulcer – made up 1% of the oral lesions present in the patients examined. 

## Discussion

A majority of the patients examined had oral lesions, of which traumatic ulcers were the most predominant. Shulman et al. [6] examined the prevalence of oral mucosal lesions in children and youths in the USA and found that a majority of them had lesions, with the lip being the most common site and lip bite/cheek bite the most common lesion. Males had more lesions present than did females. Hussein et al. [7] assessed the prevalence of oral lesions among the Jordanian children, finding that 47.4% had oral lesions and that there was no significant difference between genders. However, those authors deduced that the prevalence increased with age.

Ambika et al. [8] examined oral lesions in children attending an Indian public school and observed its presence in 64.11% of the sample population. The most common lesions evident were gingivitis, gingival abscess and traumatic ulcers.

Ulcerations can cause defects in the epithelium, connective tissue or both. Traumatic ulceration is a common oral mucosal lesion due to habits, malocclusion, tooth/teeth with sharp edges, mechanical/chemical/thermal injury, and vitamin deficiency. They are usually locatedon the buccal mucosa and labial mucosae, and can be solitary or multiple. They can persist for a few days or a few weeks but become painless three days after elimination of the injury, and the heal within 10 days [9].

Geographic tongue is a benign recurrent condition affecting the tongue with loss of epithelium. The etiology is unknown, and may be accompanied by a burning sensation or pain, or it can be asymptomatic. It persists for a few days to few weeks and can reappear at a different location [10]. 

Gingival inflammation in children can progress to cause destruction of the periodontium in the adult. The widely spaced teeth and a paucity of close occlusal contacts in children, increases the susceptibility to bacterial growth and provide for a wider area of destruction [11]. Abrams et al. [12] conducted a study on the prevalence of gingivitis in well- vs. malnourished children, finding no significant difference in the Plaque Index (PlI) and the Modified Gingival Index (MGI) between among well-nourished and malnourished groups or between males and females. However, there was less plaque and gingivitis among well-nourished children when examined with age percentiles.

Dentoalveolar abscesses are caused by bacteria such as Streptococci and Peptostreptococci, which first lead to pulpal necrosis that then progresses dentoalveolar abscesses. These bacteria can spread to the adjoining buccal, mandibular, submandibular, sublingual and submental spaces, causing space infection and subsequently cellulitis formation [13]. A study by Azodo et al. [14] assessed the presence of dentoalveolar abscesses among Nigerian children which showed a significant incidence of dentoalveolar abscesses among children, with the deciduous first molar being most commonly affected and untreated dental caries being the most common cause.

Ankyloglossia is a congenital anomaly in which the lingual frenulum is abnormally short. It is also termed “tongue tied”, with a 4.4% to 4.8% incidence in newborns and a male:female ratio of 3:1. It can lead to difficulty in swallowing, sucking and speech in children [15], [16], [17]. Its management includes frenectomy, lingual plasty and myofunctional training [18].

The prevalence of white lesions in children include frictional keratosis, leukoedema and linea alba. They can present as ulcers, color changes, and alterations in size and configuration of oral anatomy. Discontinuation of causative habits and removal of the causative irritant usually resolves the lesions [19].

The prevalence of eruption cysts is greatest among whites/people of generally European descent. It is a benign, soft-tissue cyst associated with erupting primary or permanent teeth, preceding the appearance of these teeth in the oral cavity [20]. They may disappear but should be treated if there is bleeding, pain or are infection. The management is drainage of the cystic contents [21].

## Conclusion

A thorough oral examination plays an important role in the identification and successful treatment of oral mucosal lesions. Since oral and systemic health are interlinked, more emphasis should be given on diagnosis and treatment of OMLs. Thus, the results of this study may increase the awareness of oral lesions, which might go unnoticed in the initial stage. Thus, a comprehensive oral examination should be mandatory for the pediatric population to identify mucosal lesions and variations thereof as early as possible to facilitate effective management.

## Notes

### Authors’ ORCID

- Ramiah V: 0009-0009-1493-772X
- Manivannan D: 0000-0002-2256-6297
- Shunmugavelu K: 0000-0001-7562-8802
- Kumar B: 0000-0003-2641-3498
- Dhinakaran EC: 0000-0003-2194- 6455

### Ethical approval 

Obtained

### Funding

None

### Competing interests

The authors declare that they have no competing interests.

## References

[1] Unur M, Bektas Kayhan K, Altop MS, Boy Metin Z, Keskin Y (2015). The prevalence of oral mucosal lesions in children:a single center study. J Istanb Univ Fac Dent.

[2] Gonsalves WC, Chi AC, Neville BW (2007). Common oral lesions: Part I. Superficial mucosal lesions. Am Fam Physician.

[3] Yáñez M, Escobar E, Oviedo C, Stillfried A, Pennacchiotti G (2016). Prevalence of oral mucosal lesions in children. Int J Odontostomat.

[4] Rioboo-Crespo Mdel R, Planells-del Pozo P, Rioboo-García R (2005). Epidemiology of the most common oral mucosal diseases in children. Med Oral Patol Oral Cir Bucal.

[5] El Toum S, Cassia A, Bouchi N, Kassab I (2018). Prevalence and Distribution of Oral Mucosal Lesions by Sex and Age Categories: A Retrospective Study of Patients Attending Lebanese School of Dentistry. Int J Dent.

[6] Shulman JD (2005). Prevalence of oral mucosal lesions in children and youths in the USA. Int J Paediatr Dent.

[7] Hussein AA, Darwazeh AM, Al-Jundi SH (2017). Prevalence of oral lesions among Jordanian children. Saudi J Oral Sci.

[8] Ambika L, Keluskar V, Hugar S, Ghizoni JS (2011). Prevalence of oral mucosal lesions and variations in Indian public school children. Brazil J Oral Sci.

[9] Mortazavi H, Safi Y, Baharvand M, Rahmani S (2016). Diagnostic Features of Common Oral Ulcerative Lesions: An Updated Decision Tree. Int J Dent.

[10] Nandini DB, Bhavana SB, Deepak BS, Ashwini R (2016). Paediatric Geographic Tongue: A Case Report, Review and Recent Updates. J Clin Diagn Res.

[11] Pari A, Ilango P, Subbareddy V, Katamreddy V, Parthasarthy H (2014). Gingival diseases in childhood - a review. J Clin Diagn Res.

[12] Abrams RG, Romberg E (1999). Gingivitis in children with malnutrition. J Clin Pediatr Dent.

[13] Junqueira MA, Cunha NN, Costa e Silva LL, Araújo LB, Moretti AB, Couto Filho CE, Sakai VT (2014). Surgical techniques for the treatment of ankyloglossia in children: a case series. J Appl Oral Sci.

[14] Azodo CC, Chukwumah NM, Ezeja EB (2012). Dentoalveolar abscess among children attending a dental clinic in Nigeria. Odontostomatol Trop.

[15] Messner AH, Lalakea ML, Aby J, Macmahon J, Bair E (2000). Ankyloglossia: incidence and associated feeding difficulties. Arch Otolaryngol Head Neck Surg.

[16] Friend GW, Harris EF, Mincer HH, Fong TL, Carruth KR (1990). Oral anomalies in the neonate, by race and gender, in an urban setting. Pediatr Dent.

[17] Ferrés-Amat E, Pastor-Vera T, Ferrés-Amat E, Mareque-Bueno J, Prats-Armengol J, Ferrés-Padró E (2016). Multidisciplinary management of ankyloglossia in childhood. Treatment of 101 cases. A protocol. Med Oral Patol Oral Cir Bucal.

[18] Pinto A, Haberland CM, Baker S (2014). Pediatric soft tissue oral lesions. Dent Clin North Am.

[19] Nagaveni NB, Umashankara KV, Radhika NB, Maj Satisha TS (2011). Eruption cyst: a literature review and four case reports. Indian J Dent Res.

[20] Dhawan P, Kochhar GK, Chachra S, Advani S (2012). Eruption cysts: A series of two cases. Dent Res J (Isfahan).

[21] Kassebaum NJ, Smith AGC, Bernabé E, Fleming TD, Reynolds AE, Vos T, Murray CJL, Marcenes W, GBD 2015 Oral Health Collaborators (2017). Global, Regional, and National Prevalence, Incidence, and Disability-Adjusted Life Years for Oral Conditions for 195 Countries, 1990-2015: A Systematic Analysis for the Global Burden of Diseases, Injuries, and Risk Factors. J Dent Res.

